# Bacterial Nanocellulose-Based Active Packaging for Vapor-Phase Delivery of Cinnamaldehyde to Control Fungal Spoilage in Bread

**DOI:** 10.3390/molecules31132199

**Published:** 2026-06-23

**Authors:** Érika Leão Ajala Caetano, Joana Garrossino Magalhães, Nicolli Carriel de Souza, Jair Vaz Nogueira Junior, Angela Faustino Jozala, Denise Grotto

**Affiliations:** 1 LAPETOX—Laboratory of Toxicological Research, University of Sorocaba, Sorocaba 18023-000, SP, Brazil; erikaleaoac@gmail.com (É.L.A.C.);; 2 LAMINFE—Laboratory of Industrial Microbiology and Fermentation Processes, University of Sorocaba, Sorocaba 18023-000, SP, Brazil

**Keywords:** food packaging, vapor-phase delivery, bacterial nanocellulose, cinnamaldehyde, food preservation, shelf-life

## Abstract

Active packaging systems have emerged as a promising strategy to control microbial spoilage without direct incorporation of preservatives into food matrices. In this context, this study evaluated bacterial nanocellulose (BNC) as a nanostructured carrier for vapor-phase delivery of natural antifungal compounds in bread preservation. Cinnamaldehyde (CIN), cinnamon extract and clove extract were screened against *Aspergillus niger*, *Penicillium chrysogenum*, and *Rhizopus microsporus* using minimum inhibitory concentration (MIC) and inverted halo assays. CIN demonstrated complete fungal inhibition at 0.19% (*v*/*v*), corresponding to approximately 2.0 mg/mL, outperforming plant extracts, which exhibited limited and concentration-dependent activity. When incorporated into BNC at a 1:1 ratio (50% reduced loading), CIN maintained inhibition halos comparable to the free compound, indicating effective release and preserved bioavailability. The performance of the system was further evaluated in a bread model using a non-contact active packaging approach. Fungal growth in control samples was detected by day 6 (>10^5^ CFU/g), while incorporation of plant extracts into BNC delayed spoilage to day 9 (≈50% shelf-life extension). In contrast, breads treated with CIN, either free or BNC-incorporated, showed no detectable fungal growth throughout 21 days of storage, corresponding to a shelf-life extension of at least 15 days. These results demonstrate that antifungal efficacy in vapor-phase systems depends primarily on the intrinsic potency of the active compound, while BNC acts as an effective carrier matrix that promotes sustained retention and functional availability of CIN. The use of BNC-based active packaging for cinnamaldehyde delivery represents a promising clean-label strategy to control fungal spoilage and extend the shelf life of bread without direct incorporation into the food matrix.

## 1. Introduction

Fungal contamination is one of the main factors limiting the shelf life and safety of bread and other bakery products. In recent years, active packaging systems have emerged as a promising strategy to control microbial spoilage by enabling the release of antimicrobial compounds without direct incorporation into food matrices. Species of *Aspergillus*, *Penicillium*, and *Rhizopus* are frequently associated with visible mold growth and potential mycotoxin production, leading to economic losses and food safety concerns [1,2]. Traditionally, calcium propionate and other synthetic preservatives have been incorporated into bakery formulations to control fungal proliferation. However, the prolonged consumption of synthetic additives has raised concerns regarding potential adverse health effects, including metabolic disturbances and possible bioaccumulation [3]. These concerns, combined with increasing consumer demand for minimally processed and clean-label foods, have intensified the search for natural preservation strategies.

Essential oils and plant extracts have been widely investigated due to their antimicrobial and antioxidant properties. Polysaccharide-based films incorporating essential oils have demonstrated the capacity to extend bread shelf life [4]. Among essential oil components, CIN has shown broad-spectrum antifungal activity through disruption of fungal cell membranes and interference with intracellular metabolic processes [5].

Nevertheless, the direct application of volatile compounds in food systems presents technological challenges, including rapid evaporation, reduced stability during storage, and potential sensory alterations when incorporated directly into food matrices [6]. Encapsulation and controlled-delivery systems have emerged as promising strategies to improve compound retention and prolong antimicrobial effectiveness over time [7,8].

In this context, bacterial nanocellulose (BNC) is particularly attractive due to its porous nanofibrillar structure, high surface area, and capacity to retain and gradually diffuse bioactive compounds [9]. Incorporation of cinnamaldehyde into the BNC matrix was proposed to overcome limitations related to volatility and direct food contact, allowing the active compound to be released into the package headspace while maintaining antifungal activity and reducing interaction with the bread matrix. Previous work from our group demonstrated that BNC can be used as a carrier for CIN incorporated directly into the bread matrix, resulting in effective fungal inhibition and extended shelf life [10]. However, despite its strong antifungal activity, the practical application of cinnamaldehyde in food preservation may be limited by its high volatility, susceptibility to degradation, and potential sensory impact when directly incorporated into food matrices, including changes in aroma and flavor that may affect consumer acceptance [5,6]. Consequently, delivery systems capable of retaining compound and supporting their gradual release have attracted increasing interest as strategies to preserve functional performance while minimizing undesirable effects [7,8].

In this context, vapor-phase delivery through active packaging represents a promising alternative, as it enables antifungal control without direct contact between the active compounds and the food product. Bacterial nanocellulose is particularly attractive for this purpose due to its porous nanofibrillar structure, high surface area, and ability to retain bioactive compounds [9]. To date, limited information is available regarding the use of bacterial nanocellulose as a carrier in vapor-phase antifungal packaging systems for bread preservation. Therefore, the present study advances previous findings by shifting from direct incorporation of CIN into the food matrix to a non-contact active packaging approach, in which the active compounds are delivered through the package headspace. This strategy was investigated as an alternative to preserve antifungal efficacy while minimizing direct interaction with the food product.

Therefore, this study aimed to evaluate selected natural antifungal compounds and investigate the effectiveness of a BNC-based active packaging system for vapor-phase delivery as an alternative strategy to control fungal growth in bread without direct incorporation into the food matrix.

## 2. Results and Discussion

### 2.1. Nanoparticle Characterization

Nanoparticle Tracking Analysis revealed that the BNC dispersion presented an average hydrodynamic diameter of 18.5 nm and a particle concentration of 3.64 × 10^8^ particles/mL, confirming its classification within the nanomaterial range (<100 nm in at least one dimension). These values are consistent with the expected dimensions of enzymatically disintegrated bacterial cellulose nanofibrils.

Bacterial cellulose is intrinsically composed of ultrafine, highly crystalline nanofibrillar networks, typically ranging from 10 to 100 nm in diameter, which confer high surface area and structural uniformity compared to plant-derived cellulose [9]. The nanometric scale and elevated particle concentration observed in the present study are particularly relevant for the functional performance of the system, as they increase the available interfacial area for interaction with bioactive compounds and favor controlled diffusion.

This structural configuration likely contributed to the efficient retention and release behavior observed for CIN, supporting the maintenance of antifungal activity even at reduced loading levels.

NTA enables individual particle tracking based on Brownian motion and calculates hydrodynamic diameter using the Stokes–Einstein equation [11]. Compared to techniques such as dynamic light scattering, NTA provides more reliable analysis of polydisperse suspensions, which is particularly relevant for nanocellulose dispersions containing heterogeneous fibrillar fragments.

The nanoscale size distribution observed supports a high available surface area and abundant hydroxyl groups, which favor interaction with incorporated bioactive compounds [9]. In addition, NTA revealed a particle concentration of 3.64 × 10^8^ particles/mL. Particle concentration is a critical parameter in nanodelivery systems, as it influences loading capacity and dispersion stability [12].

### 2.2. Determination of Cinnamaldehyde Retention by UV–Vis Spectrophotometry

The retention of CIN within the BNC matrix was evaluated over a 21-day storage period by UV–Vis spectrophotometry. The initial concentration (day 1) was approximately 740 mg/mL, decreasing to approximately 637 mg/mL after 21 days of storage, corresponding to a reduction of only 14%.

These results indicate that the BNC matrix is capable of retaining a substantial fraction of CIN over time, despite its inherent volatility. The limited reduction observed suggests that BNC acts as a protective environment, reducing rapid evaporation and promoting a more sustained release of the active compound.

The retention of more than 85% of the initial cinnamaldehyde content throughout the evaluation period is particularly relevant because the antifungal activity observed in both halo assays and bread preservation experiments was maintained over the same timeframe. These findings support the hypothesis that BNC functions not only as a retention matrix but also as a delivery system, preserving the functional availability of cinnamaldehyde while allowing its gradual diffusion into the surrounding environment.

Although release kinetics were not directly measured, the combination of quantitative retention data and sustained antifungal performance suggests that cinnamaldehyde remained available at biologically effective concentrations throughout storage.

This behavior is consistent with polymer-based delivery systems described in the literature, in which release is governed by concentration gradients and reversible physical interactions between the active compound and the polymeric network [13]. In the case of BNC, hydrogen bonding and van der Waals interactions within the highly porous nanofibrillar structure may contribute to the temporary retention of cinnamaldehyde without preventing its subsequent diffusion and antifungal action.

This mechanism may explain the prolonged preservation effect observed in bread samples during storage.

### 2.3. Minimum Inhibitory Concentration (MIC)

CIN exhibited complete inhibition of *Aspergillus niger*, *Rhizopus microsporus*, and *Penicillium chrysogenum* at all tested concentrations, including the lowest concentration evaluated (0.19% (*v*/*v*), corresponding to approximately 2.0 mg/mL). In contrast, the hydroalcoholic extracts showed limited and concentration-dependent activity. For all fungal species, growth was observed at relatively high extract concentrations, indicating lower antifungal potency under the experimental conditions. This result highlights the high antifungal potency of CIN against common bakery spoilage fungi.

The superior performance of CIN is consistent with its well-documented membrane-disruptive mechanism. As a low-molecular-weight aldehyde, it readily interacts with fungal membrane lipids, alters membrane permeability, and interferes with ergosterol-associated structural organization, leading to metabolic dysfunction and growth inhibition [1,2,5].

In contrast, crude extracts comprise complex phytochemical matrices in which the effective concentration of active antifungal constituents is reduced and potentially influenced by compound–compound interactions [14]. This compositional variability likely explains the incomplete inhibition observed for clove and cinnamon extracts.

### 2.4. Inverted Halo Assay and Stability

**Figure 1:**
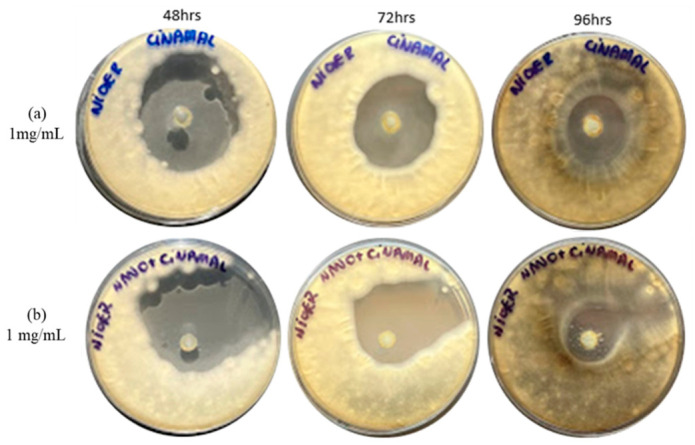
Inverted halo assay for *Aspergillus niger*. (**a**) Cinnamaldehyde (CIN); (**b**) Cinnamaldehyde incorporated into bacterial nanocellulose (BNC).

**Figure 2:**
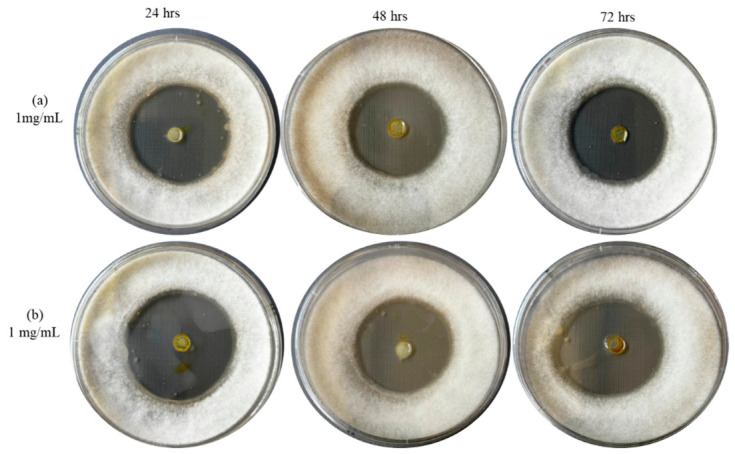
Inverted halo assay for *Rhizopus microsporus*. (**a**) Cinnamaldehyde (CIN); (**b**) Cinnamaldehyde incorporated into bacterial nanocellulose (BNC).

**Figure 3:**
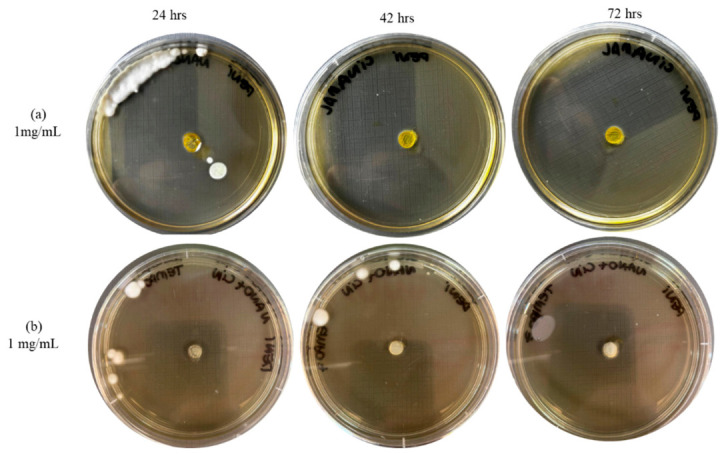
Inverted halo assay for *Penicillium chrysogenum*. (**a**) Cinnamaldehyde (CIN); (**b**) Cinnamaldehyde incorporated into bacterial nanocellulose (BNC).

As evidenced in Figure 1, Figure 2 and Figure 3, CIN maintained clearly defined inhibition halos against *Aspergillus niger*, Rhizopus microsporus, and *Penicillium chrysogenum*, both in its free form and after incorporation into BNC. Of particular relevance is the observation that the incorporated formulation (Figure 1b, Figure 2b and Figure 3b), containing approximately 50% of the original CIN concentration, exhibited halo diameters comparable to those observed for the pure compound.

This behavior suggests that incorporation into BNC preserved antifungal activity while improving the efficiency of active compound utilization. The preservation of inhibition halo diameter after incorporation is particularly relevant because it indicates that BNC maintained the functional availability of cinnamaldehyde while allowing efficient diffusion through the matrix. This suggests that the nanocellulose network acted as a carrier system rather than a barrier, preserving the antifungal effectiveness of the compound despite the reduced loading. In solid diffusion systems, halo formation is governed by the relationship between the radial concentration gradient and the minimum inhibitory concentration (MIC). When a compound exhibits a low MIC, as previously observed for CIN (0.19%), small released amounts are sufficient to reach the inhibitory threshold at the periphery of the diffusion zone [15]. Therefore, the maintenance of halo diameter despite reduced loading indicates that BNC enabled effective release of the compound without excessive retention or diffusion limitation.

The three-dimensional nanofibrillar structure of BNC is characterized by high porosity, large specific surface area, and an interconnected network of microfibrils. Similar systems have been described as matrices capable of functioning simultaneously as physical reservoirs and modulators of diffusional release of bioactive compounds [16]. Retention of the active compound occurs predominantly through physical interactions, including hydrogen bonding, van der Waals interactions, and capillary retention within the matrix, allowing diffusion to be governed by concentration gradients rather than irreversible entrapment [17]. These interactions are not expected to irreversibly trap the compound, but rather to temporarily retain it and support its gradual release over time. In the active packaging system, CIN release is primarily governed by diffusion from the BNC matrix into the package headspace, followed by vapor-phase distribution and subsequent interaction with fungal cells.

Furthermore, incorporation into polymeric matrices has been reported as an effective strategy to reduce volatilization and oxidative degradation of phenolic compounds, thereby enhancing their stability and functional availability [18]. Considering the volatile nature of CIN, BNC may have acted as a protective microenvironment, minimizing immediate losses and promoting more sustained release throughout the assay. This hypothesis consists of polymer-based systems containing essential oils, in which antimicrobial activity depends on the fraction of active compound effectively released and available for diffusion [8].

In contrast, clove and cinnamon extracts did not exhibit inhibition halos, either in free form or after incorporation into BNC. This outcome should be interpreted in light of two primary factors: intrinsic antifungal potency and molar availability of the active principle. Plant extracts constitute complex phytochemical systems in which the major active compound is diluted among various secondary constituents, reducing the effective concentration available [19]. Comparative studies demonstrate that isolated phenolic compounds exhibit significantly higher antifungal activity than crude extracts due to greater molar availability and absence of matrix interference [20].

Additionally, reviews addressing cinnamon applications in antimicrobial systems emphasize that antifungal efficacy is directly correlated with the cinnamaldehyde content present in the formulation [4]. When used as an extract, the effective concentration may be insufficient to reach the local MIC after radial diffusion in agar. Thus, even if partial antifungal activity exists, the combination of high MIC and insufficient concentration gradient prevents the formation of measurable inhibition halos.

Incorporation into BNC did not enhance the intrinsic biological activity of the extracts, confirming that the matrix acts as a delivery system rather than an activity enhancer. While release modulation cannot compensate for low intrinsic potency, it can improve functional efficiency when highly active compounds such as CIN are used, allowing maintenance of antifungal activity even at reduced loading.

In Table 1, the stability profile of CIN incorporated into BNC exhibited fungus-dependent behavior against common bakery spoilage fungi. For *Aspergillus niger*, a significant reduction in inhibition halo diameter was observed at 48 and 96 h compared to free CIN, indicating a partial decline in antifungal efficacy over time. This decrease may be attributed to the gradual diffusion and volatilization of CIN from the nanocellulosic matrix, a behavior commonly reported for phenolic aldehydes due to their high vapor pressure and chemical reactivity.

Polymeric matrices, such as bacterial cellulose, are described as porous nanofibrillar networks capable of modulating release kinetics through diffusion-controlled mechanisms [16]. Although such systems can reduce immediate volatilization and oxidative degradation [19], sustained release may result in a lower instantaneous concentration available at later time points, particularly against fungi with higher resistance profiles, such as *A. niger*. This may explain the statistically significant reduction observed for the incorporated formulation at extended incubation periods.

In contrast, for *Rhizopus microsporus*, no statistically significant differences were observed between free CIN and the incorporated formulation across the evaluated time points. This suggests that the concentration released from the BNC matrix remained above the inhibitory threshold for this species. The maintenance of antifungal activity indicates that the nanocellulose matrix effectively preserved functional availability of CIN during the initial stages of storage.

These findings reinforce that the performance of nanocellulose-based delivery systems depends on the interaction between intrinsic compound potency, release kinetics, and fungal susceptibility. While incorporation did not enhance intrinsic antifungal activity, it allowed maintenance of biologically effective concentrations over time, particularly against *R. microsporus*, supporting the feasibility of BNC as a carrier system for volatile antifungal agents.

### 2.5. Application in Bread Using Vapor-Phase Active Packaging

**Figure 4:**
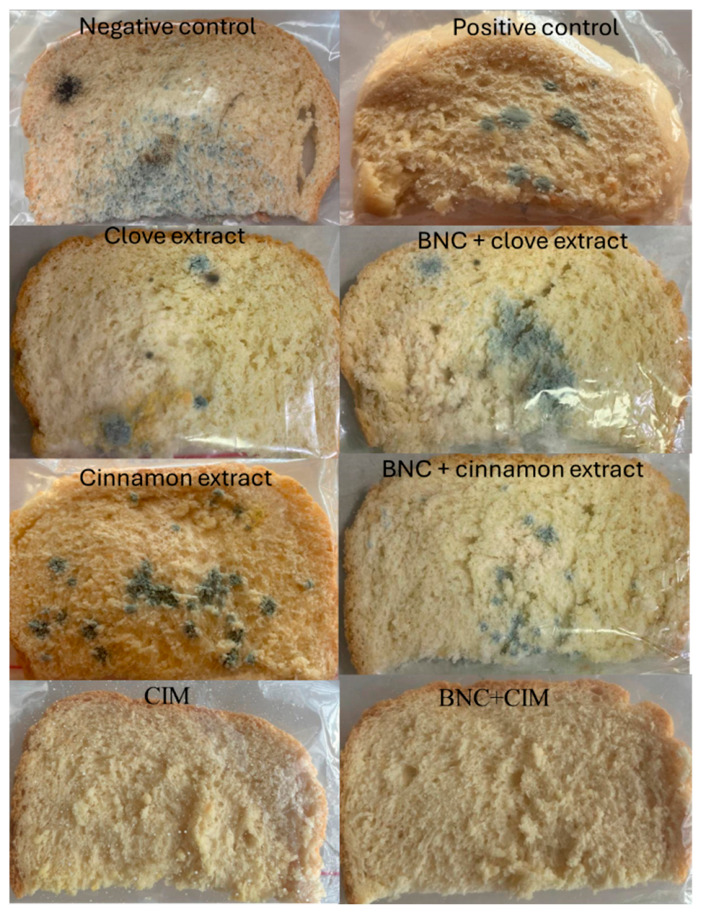
Fungal growth in bread treated under vapor-phase active packaging conditions with natural antifungal compounds, with and without bacterial nanocellulose (BNC).

As shown in Table 2 and Figure 4, fungal growth was detected on day 6 in both the negative control and the positive control, indicating limited protection under the storage conditions evaluated in this study. Similar onset of growth was observed in breads treated with clove and cinnamon extracts, confirming the low antifungal efficacy previously demonstrated in the MIC and halo assays.

Incorporation of the extracts into BNC delayed visible fungal growth from day 6 to day 9, corresponding to a shelf-life extension of approximately three days (≈50%) compared to the control groups. This improvement suggests that the nanocellulose matrix contributed to moderated release of bioactive compounds within the sealed packaging system. Bacterial cellulose has been described as a porous nanofibrillar network capable of acting as a reservoir and diffusion regulator for volatile antimicrobial agents [16]. Controlled release systems based on polysaccharide matrices have been shown to prolong bread shelf life by approximately 2–4 days when combined with essential oils [8], which aligns with the delay in fungal growth observed in the present study.

Notably, no fungal growth was observed in breads treated with CIN, either free or incorporated into BNC, throughout the 21-day evaluation period (Table 2 and Figure 4). This represents a shelf-life extension of at least 15 days compared to the controls. The strong antifungal performance aligns with previously reported activity of CIN against bakery spoilage fungi [21] and supports its effectiveness in vapor-phase applications.

In vapor-phase active packaging systems, the active compound is not incorporated directly into the food but is released from the packaging environment into the package headspace [22,23]. For volatile compounds such as CIN, antifungal activity depends on evaporation, diffusion through the headspace, and subsequent contact with fungal cells on the bread surface at concentrations above the inhibitory threshold [21,23]. This non-contact mechanism may be particularly advantageous for bakery products because fungal spoilage commonly begins at the surface, where vapor-phase compounds can exert direct inhibitory effects while minimizing interaction with the food matrix.

Compared with our previously reported direct-incorporation approach [10], the present vapor-phase strategy achieved sustained antifungal protection without direct contact between the active compound and the food matrix. This characteristic may be advantageous for bakery applications because it reduces the likelihood of sensory alterations while maintaining antifungal performance.

Importantly, incorporation into BNC did not reduce the protective effect of CIN, indicating that the nanocellulose matrix preserved functional availability of the compound while potentially moderating volatilization. Polymer-based active packaging systems containing essential oils have similarly demonstrated sustained antimicrobial performance and significant shelf-life extension in bread products [22].

### 2.6. Quantitative Fungal Growth Assessment

**Figure 5:**
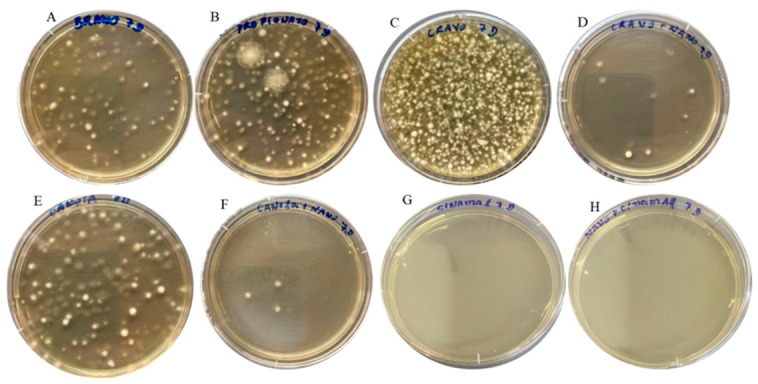
Quantitative fungal count on day 7 for bread samples. (**A**) Negative control; (**B**) Positive control; (**C**) Clove; (**D**) Clove + bacterial nanocellulose (BNC); (**E**) Cinnamon; (**F**) Cinnamon + bacterial nanocellulose (BNC); (**G**) Cinnamaldehyde (CIN); (**H**) Cinnamaldehyde (CIN) + bacterial nanocellulose (BNC).

**Figure 6:**
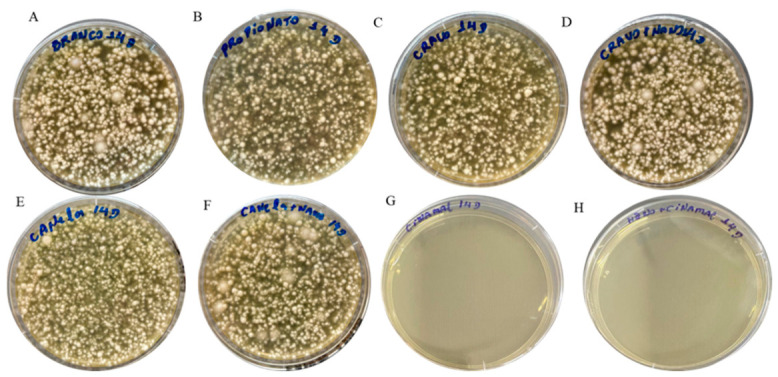
Quantitative fungal count on day 14 for bread samples. (**A**) Negative control; (**B**) Positive control; (**C**) Clove; (**D**) Clove + bacterial nanocellulose (BNC); (**E**) Cinnamon; (**F**) Cinnamon + bacterial nanocellulose (BNC); (**G**) Cinnamaldehyde (CIN); (**H**) Cinnamaldehyde (CIN) + bacterial nanocellulose (BNC).

**Figure 7:**
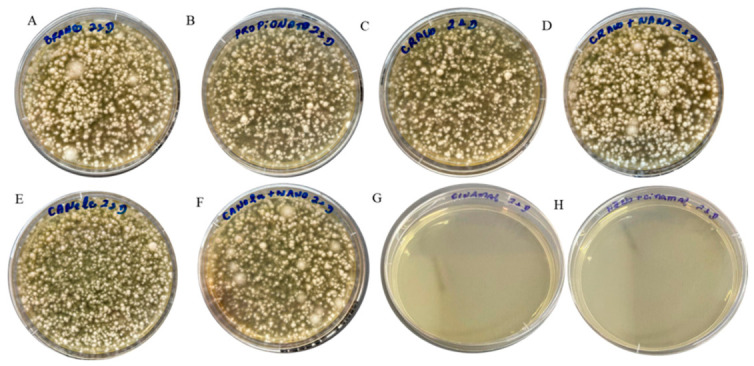
Quantitative fungal count on day 21 for bread samples. (**A**) Negative control; (**B**) Positive control; (**C**) Clove; (**D**) Clove + bacterial nanocellulose (BNC); (**E**) Cinnamon; (**F**) Cinnamon + bacterial nanocellulose (BNC); (**G**) Cinnamaldehyde (CIN); (**H**) Cinnamaldehyde (CIN) + bacterial nanocellulose (BNC).

Quantitative fungal counts (Figure 5, Figure 6 and Figure 7) confirmed the visual results. Both negative and positive controls showed fungal growth by day 6, exceeding 10^5^ CFU/g by day 7, indicating limited protection under the tested conditions. Similar behavior was observed for breads treated with clove and cinnamon extracts alone, consistent with their previously observed high MIC values and lack of inhibition halos. Crude extracts often show reduced efficacy in food matrices due to low effective concentration of active compounds and interactions within complex systems [1,20]. In complex food systems, antifungal efficacy observed in vitro may not be fully reproduced due to several matrix-related factors, including water activity, pH, fat and carbohydrate content, food structure, compound partitioning, volatility, interactions with proteins or starch, and heterogeneous diffusion [5,6]. These factors can reduce the effective concentration of active compounds at the fungal growth site and directly affect antimicrobial performance. This is particularly relevant for plant extracts, in which active constituents are present at lower concentrations and may be further influenced by interactions with food matrix components [19,20].

Incorporation of clove and cinnamon extracts into BNC delayed visible spoilage from day 6 to day 9, corresponding to a shelf-life extension of approximately three days. On day 7, fungal counts remained below the Brazilian regulatory limit (5 × 10^4^ CFU/g). This short-term improvement is consistent with reports that polysaccharide-based delivery systems can temporarily enhance antifungal performance through controlled release of volatile compounds [8,16]. However, by day 14, fungal growth became uncountable, suggesting depletion of active compounds or insufficient sustained release.

In contrast, breads treated with CIN, either free or incorporated into BNC, showed no fungal growth throughout 21-day storage period, representing a shelf-life extension of at least 15 days compared to the control groups. This sustained inhibition aligns with well-established antifungal mechanism of CIN [1,2]. Importantly, BNC incorporation did not reduce its efficacy, indicating that the BNC matrix preserved functional availability of the compound while potentially moderating volatilization [18].

Overall, shelf-life extension in this system was primarily driven by intrinsic antifungal potency, with BNC acting as an effective delivery platform when combined with a highly active compound such as CIN, enabling improved control of fungal spoilage in bread.

This study represents an advancement over previous approaches by transitioning from direct incorporation into the food matrix to a vapor-phase active packaging system, while also providing quantitative evidence of CIN retention over time. From an application perspective, vapor-phase delivery systems may be more suitable for industrial implementation, as they minimize formulation constraints and potential alterations in product sensory attributes. By avoiding direct addition of volatile compounds to the food matrix, this approach may contribute to improved consumer acceptability while maintaining antifungal efficacy. Nevertheless, dedicated sensory studies are required to confirm the impact of this strategy on aroma, flavor, and overall consumer perception.

## 3. Materials and Methods

### 3.1. Production of Bacterial Nanocellulose (BNC)

Bacterial nanocellulose (BNC) was biosynthesized using *Komagataeibacter xylinus* (ATCC 53582). The pre-inoculum was prepared in Hestrin and Schramm (HS) liquid medium containing 20 g/L glucose, 5 g/L yeast extract, 1.15 g/L Na_2_HPO_4_ (anhydrous), and 1.15 g/L citric acid monohydrated. Cultures were incubated statically at 30 °C for 48 h to allow bacterial adaptation and initial growth. For cellulose production, 10% (*v*/*v*) of the pre-inoculum was transferred to sterile plastic containers containing fresh HS medium. Static fermentation was carried out at 30 °C for 10 days, allowing the formation of cellulose pellicles at the air–liquid interface.

The resulting cellulose membranes were purified by alkaline treatment in 1 M NaOH at 60 °C for 90 min to remove bacterial biomass and residual culture components. The membranes were subsequently washed with distilled water until neutral pH was achieved, following the protocol [23].

Purified cellulose pellicles were mechanically disintegrated using an industrial blender and further homogenized using an Ultra-Turrax^®^ disperser (IKA, Staufen, Germany) for 5 min to reduce fibril aggregation and obtain a uniform suspension. The resulting dispersion was filtered through a fine mesh strainer to remove excess water.

To obtain nanostructured cellulose, 5 g of the blended cellulose were suspended in 15 mL of Milli-Q water obtained from a Milli-Q purification system (Merck Millipore, Burlington, MA, USA) and heated to 50 °C under constant magnetic stirring. Enzymatic hydrolysis was initiated by adding 500 µL of cellulase (≥700 U/g, from *Trichoderma reesei*, Sigma-Aldrich, St. Louis, MO, USA). The reaction was maintained for 30 min under continuous stirring. Enzyme inactivation was performed by heating the suspension to 100 °C for 20 min.

The suspension was subsequently centrifuged at 5500 rpm for 20 min using a Sorvall™ ST 16R centrifuge (Thermo Fisher Scientific, Waltham, MA, USA), and the supernatant containing dispersed BNC nanoparticles was collected. This procedure was adapted [24], with modifications to optimize nanoparticle dispersion.

### 3.2. Nanoparticle Characterization

The bacterial nanocellulose (BNC) dispersion was analyzed by Nanoparticle Tracking Analysis (NTA) using a NanoSight PRO platform (Malvern Instruments, Malvern, UK) operated with NS Xplorer software (v1.2.0.3). Prior to analysis, samples were diluted 1:100 in ultrapure water to ensure particle concentrations within the recommended detection interval (10^6^–10^9^ particles/mL).

Measurements were carried out at 25 °C, and five independent 60 s recordings were collected for each sample to enhance analytical robustness. Individual particle movement resulting from Brownian motion was monitored and processed by the software to calculate hydrodynamic diameter (mean and modal values), size distribution profile, and particle number concentration (particles/mL), based on diffusion coefficients derived from the Stokes–Einstein relationship.

NTA is widely recognized as a reliable technique for the characterization of heterogeneous nanoparticle dispersions and has been extensively applied to cellulose-based nanomaterials due to its ability to simultaneously determine particle size distribution and concentration [25].

### 3.3. Incorporation of Natural Active Compounds into BNC

Cinnamaldehyde (CIN) oil, cinnamon extract, and clove extract were incorporated into the BNC dispersion at a 1:1 (*v*/*v*) ratio in sterile polypropylene Falcon^®^ tubes. The mixtures were subjected to orbital agitation (NT 715 shaker, Nova Técnica, Piracicaba, Brazil) at 25 °C for 4 h at 100 rpm to promote homogeneous dispersion and interaction between the nanocellulose matrix and the bioactive compounds. The procedure was adapted [26], with minor modifications.

CIN (≥98% purity) was purchased from Vidara in São Paulo, Brazil. The cinnamon extract consisted of a 20% (*w*/*v*) hydroalcoholic tincture obtained from the bark of *Cinnamomum zeylanicum*, produced by percolation/maceration in a 56° ethanol–water solution and supplied by Botica Alternativa Homeopatia Ltd.a (Curitiba, Brazil). Clove extract was used under the same commercial specifications.

### 3.4. Determination of Cinnamaldehyde Retention by UV–Vis Spectrophotometry

The concentration of CIN retained in the BNC matrix was determined by UV–Vis spectrophotometry. Samples were collected after 1 and 21 days of storage under controlled conditions.

Prior to analysis, samples were diluted in methanol to ensure absorbance values within the linear range of the analytical method. Absorbance measurements were performed at 285 nm, corresponding to the characteristic absorption maximum of cinnamaldehyde in methanolic solution.

A calibration curve was constructed using standard cinnamaldehyde solutions of known concentration, and sample concentrations were calculated from the resulting linear regression equation after correction for the dilution factor.

### 3.5. Determination of Minimum Inhibitory Concentration (MIC)

The minimum inhibitory concentration (MIC) of cinnamon extract, clove extract, and CIN oil was determined using reference fungal strains associated with bakery spoilage: *Aspergillus niger* (ATCC 5275), *Penicillium chrysogenum* (ATCC 48905), and *Rhizopus microsporus* var. *oligosporus* (ATCC 22959).

MIC assays were performed in sterile 96-well microplates. Each well received 100 µL of Sabouraud dextrose broth (SDB), followed by 10 µL of standardized fungal inoculum (10^6^ CFU/mL) and 100 µL of the test compound, added sequentially under aseptic conditions.

Cinnamon and clove extracts were evaluated at an initial concentration of 20% (*v*/*v*), whereas CIN was tested at 100% purity. The lowest concentration showing complete inhibition corresponded to 0.19% (*v*/*v*), equivalent to approximately 2.0 g/L based on the density of cinnamaldehyde (1.05 g/mL). Negative controls (broth without inoculum) and positive controls (inoculated broth without antimicrobial compound) were included in each plate to confirm sterility and fungal viability, respectively.

Microplates were incubated at 25 °C for 72 h under static conditions. To minimize volatilization of CIN oil and prevent cross-well vapor diffusion, plates were immediately sealed with Parafilm^®^ after addition of the inoculum and test compounds. This precaution reduced potential headspace interference and improved reliability of MIC determination for volatile compounds [27].

After incubation, fungal growth was evaluated by transferring 10 µL from each well onto Sabouraud dextrose agar plates, followed by incubation at 25 °C. The MIC was defined as the lowest concentration of the tested compound that completely inhibited visible fungal growth.

### 3.6. Inverted Halo Assay and Stability

The antifungal activity of BNC-incorporated formulations was assessed using the inverted halo method on Sabouraud dextrose agar plates. A 200 µL aliquot of fungal suspension (10^6^ CFU/mL) was evenly spread across the agar surface. Wells (10 mm diameter) were aseptically perforated at the center of each plate, as previously described [28].

Each well was filled with 20 µL of either the free active compound or the corresponding BNC-incorporated formulation. Incorporation was performed at a 1:1 (*v*/*v*) ratio, resulting in final concentrations of 10% for cinnamon extract and clove extract, and 50% for CIN oil. These concentrations were established through preliminary optimization to ensure formulation stability and homogeneous dispersion.

Because CIN is a purified compound with inherently higher antimicrobial potency than crude cinnamon and clove extracts, the objective was not to perform a molar comparison between treatments. Instead, the assay was designed to evaluate the suitability of BNC as a delivery matrix for both isolated bioactive compounds and complex plant extracts under standardized experimental conditions. For this reason, the same CIN concentration was maintained across all fungal species to allow direct comparison between the free and BNC-incorporated formulations and to evaluate the release performance of the nanocellulose-based system.

Plates were incubated at 25 ± 1 °C for 96 h. Because inhibition zones produced by volatile compounds against filamentous fungi were not always perfectly circular, halo diameters were measured daily at four equidistant points passing through the center of the well using a digital caliper, and the mean diameter was calculated. The irregular halo contours were likely associated with the volatility and radial diffusion behavior of cinnamaldehyde in the agar medium, the gradual release of the compound from the nanofibrillar BNC matrix, and the intrinsically non-uniform radial growth pattern of filamentous fungi on solid media. All experiments were performed in triplicate [27]. The stability of the BNC-incorporated formulations was monitored throughout the experimental period to ensure maintenance of dispersion and antifungal activity.

### 3.7. Application in Bread Using Vapor-Phase Active Packaging

Bread loaves (500 ± 30 g) were prepared using 288.5 g of wheat flour, 5.5 g of powdered milk, 14 g of sugar, 5 g of salt, 8.5 g of margarine, 85 g of fresh yeast, and 170 mL of warm water. Dough preparation and handling were conducted under aseptic conditions inside a laminar flow hood. The mixture was processed in a domestic bread machine (Master Bread NPF-53, Mondial, Conceição do Jacuípe, BA, Brazil) using the “quick” program (1 h 55 min).

After baking, the loaves were cooled and sliced into 5 cm thick portions under aseptic conditions. All treatments were performed in triplicate.

Active formulations consisting of clove extract, cinnamon extract, and CIN incorporated into BNC were applied through a non-contact active packaging system. Polyethylene bags were sterilized under UV light for 60 min prior to use. Subsequently, 500 µL of each BNC-based formulation were sprayed onto the internal surface of the bags before bread insertion. The bags were briefly equilibrated to allow uniform distribution of the active material and then sealed.

The experimental groups consisted of: (i) negative control (bread without preservative), (ii) positive control (bread containing 0.1% calcium propionate, according to ANVISA guidelines), (iii) bread with clove extract, (iv) bread with BNC + clove extract, (v) bread with cinnamon extract, (vi) bread with BNC + cinnamon extract, (vii) bread with CIN, and (viii) bread with BNC + CIN.

A treatment consisting solely of BNC was not included as an independent experimental group because preliminary in vitro assays (MIC and halo tests) demonstrated that BNC alone exhibited no antifungal activity. As a structurally inert polysaccharide matrix, bacterial nanocellulose functions exclusively as a delivery system and does not possess intrinsic antimicrobial properties, as consistently reported in the literature [16].

No antimicrobial agents were incorporated directly into the bread matrix. Therefore, fungal inhibition occurs exclusively by vapor-phase diffusion within the sealed packaging environment. The amount of CIN used in the formulations was established based on the Acceptable Daily Intake (ADI) of 1.25 mg/kg body weight/day [29]. This approach ensured that the levels used in the packaging system remained within internationally recognized safety limits.

All samples were stored under controlled laboratory conditions, and fungal growth was evaluated daily by visual inspection. The levels of CIN used in the packaging system were established based on internationally recognized safety limits. This approach enables antifungal activity without direct contact between the active compound and the food matrix.

### 3.8. Quantitative Fungal Growth Assessment

Fungal enumeration was performed on days 7, 14, and 21 after bread slices were placed into the polypropylene packaging previously sprayed with the antimicrobial formulations. Analyses were performed in triplicate following a protocol adapted [30].

For each sampling point, 25 g of bread were aseptically collected, macerated, and homogenized in 225 mL of 0.1% peptone water to obtain an initial 10^−1^ dilution. Subsequent serial tenfold dilutions (10^−2^ and 10^−3^) were prepared under sterile conditions. Aliquots (1 mL) from each dilution were plated onto Sabouraud soft agar.

Plates were incubated at 25 ± 1 °C for up to 48 h, after which colony-forming units were counted and expressed as CFU/g of sample.

### 3.9. Statistical Analysis

For the stability test, comparisons between the CIN and NCIN treatments were performed using Student’s *t*-test. Statistical significance was set at *p* < 0.05.

## 4. Conclusions

The present study demonstrates a shift from in-matrix delivery to packaging-based delivery systems as a strategy to overcome limitations associated with direct incorporation of volatile antifungal compounds. Although clove and cinnamon extracts exhibited limited standalone efficacy, their incorporation into BNC temporarily delayed fungal growth, extending shelf life by approximately three days. This confirms that BNC can modulate release kinetics and improve short-term protection, but cannot compensate for low intrinsic antifungal potency.

In contrast, CIN showed robust and sustained antifungal activity, completely inhibiting fungal growth for at least 21 days, indicating a shelf-life extension of more than two weeks compared to untreated bread. Notably, incorporation into BNC preserved this efficacy even at reduced loading, demonstrating that the nanocellulose matrix promoted sustained retention and maintained the functional availability of the compound throughout storage without impairing antifungal activity.

Collectively, these findings indicate that CIN delivered via BNC represents a promising natural alternative to synthetic preservatives for controlling fungal spoilage in bakery products. The combination of high intrinsic antifungal potency and nanostructured delivery highlights the potential of bacterial nanocellulose-based active packaging systems for sustainable shelf-life extension in bread products. Beyond bread preservation, the proposed BNC-based active packaging system may also be applicable to other foods susceptible to surface fungal spoilage, including cakes, pastries, tortillas, cheeses, dried fruits, and other intermediate-moisture foods. Because the antifungal compound is delivered through the package headspace rather than directly incorporated into the product, this strategy may offer a versatile platform for clean-label preservation while minimizing formulation changes and potentially reducing sensory alterations associated with direct addition of volatile compounds to food matrices. Such characteristics may be particularly advantageous for bakery products, where consumer acceptance is strongly influenced by aroma and flavor attributes.

## Figures and Tables

**Table 1:** Stability of Cinnamaldehyde (CIN) and Cinnamaldehyde incorporated into bacterial nanocellulose (BNC) against *Aspergillus niger* and *Rhizopus microsporus*: inhibition halo diameters (mm) measured at different time points. Values are expressed as mean ± standard deviation (*n* = 3/group). * *p* < 0.05 compared to CIN.

	CIN	CIN + BNC
Inhibition halo diameters (mm) *Aspergillus niger*		
48 h	52.0 ± 2.2	42.0 ± 2.4 *
72 h	37.5 ± 1.2	32.5 ± 4.9
96 h	25.0 ± 0.4	21.0 ± 0 *
Inhibition halo diameters (mm) *Rhizopus microsporus*		
24 h	49.5 ± 1.2	50.7 ± 1.0
48 h	51.0 ± 4.7	52.5 ± 1.9
72 h	48.5 ± 4.4	43.5 ± 0.5

**Table 2:** Day of fungal growth for each sample and mean number of colonies visually observed. Data are presented as mean ± standard deviation (*n* = 3/group).

Sample	Day of Fungal Growth	Mean Colony Count CFU/g
Negative control	6	8.0 ± 2.0
Positive control	6	7.6 ± 2.0
Bread with clove extract	6	5.3 ± 2.0
Bread with BNC + clove extract	9	5.3 ± 1.0
Bread with cinnamon extract	6	17.0 ± 3.5
Bread with BNC + cinnamon extract	9	6.6 ± 0.6
Bread with CIN	-	-
Bread with BNC + CIN	-	-

## Data Availability

The data that support the findings of this study are available from the corresponding author upon reasonable request.
